# 

**Published:** 1896-11

**Authors:** 


### CONTENTS.

ORIGINAL ARTICLES—

An Epitome of the History of Pathology. By Charles Mynor Blackford, Jr., M. D., Atlanta, Ga ........................ 535

SOCIETY REPORTS—

Richmond Academy of Medicine and Surgery ................ 556

SELECTIONS AND ABSTRACTS—

The Non-Operative Treatment of Urethral Strictures. By Ferd. C. Valentine, M. D., ................................ 559

Why is the Negro Black ? ....... ................................ 565

SELECTIONS AND ABSTRACTS—(Continued.)

Guaiacol Externally as are Antipyretic........................ 566

The Treatment of Neuralgic and Rheumatic Affections. By D. S. Maddox, M. D.,.......................................... 567

EDITORIAL................................................ 569

NOTES.................................................... 572

SPECIAL NOTES............................................ 577

PRESCRIPTIONS............................................ 587

